# Construction of hyperbranched polylysine-modified antibacterial polyurethane foam dressings and their promotion of infected deep burn wound healing

**DOI:** 10.1093/rb/rbag178

**Published:** 2026-08-18

**Authors:** Xiaoqing Liu, Huan Liu, Bingjie Fu, Qiaoju Hu, Min Liang, Xingang Wang, Changyou Gao

**Affiliations:** Zhejiang Key Laboratory of Advanced Organic Materials and Technologies, MOE Key Laboratory of Macromolecular Synthesis and Functionalization, Department of Polymer Science and Engineering, Zhejiang University, Hangzhou 310058, China; Department of Burns and Wound Care Center, Second Affiliated Hospital, College of Medicine, Zhejiang University, Hangzhou 310009, China; Polytechnic Institute, Zhejiang University, Hangzhou 310058, China; Zhejiang Engineering Research Center for Interface Technology of Medical Polymers and Devices, Shaoxing Key Laboratory of Healthcare Materials and Application Technology, and Center for Healthcare Materials, Shaoxing Institute, Zhejiang University, Shaoxing 312099, China; Zhejiang Engineering Research Center for Interface Technology of Medical Polymers and Devices, Shaoxing Key Laboratory of Healthcare Materials and Application Technology, and Center for Healthcare Materials, Shaoxing Institute, Zhejiang University, Shaoxing 312099, China; Department of Burns and Wound Care Center, Second Affiliated Hospital, College of Medicine, Zhejiang University, Hangzhou 310009, China; Zhejiang Key Laboratory of Advanced Organic Materials and Technologies, MOE Key Laboratory of Macromolecular Synthesis and Functionalization, Department of Polymer Science and Engineering, Zhejiang University, Hangzhou 310058, China; Polytechnic Institute, Zhejiang University, Hangzhou 310058, China; Zhejiang Engineering Research Center for Interface Technology of Medical Polymers and Devices, Shaoxing Key Laboratory of Healthcare Materials and Application Technology, and Center for Healthcare Materials, Shaoxing Institute, Zhejiang University, Shaoxing 312099, China; The State Key Laboratory of Transvascular Implantation Devices, The Second Affiliated Hospital, Zhejiang University, Hangzhou 310009, China; Dr. Li Dak Sum & Yip Yio Chin Center for Stem Cell and Regenerative Medicine, Zhejiang University, Hangzhou 310058, China

**Keywords:** foam dressing, hyperbranched polylysine, antibacterial modification, biological safety, deep burn wound

## Abstract

Foam dressings are vital for exuding wounds, yet antibacterial modification remains challenging and relies heavily on cytotoxic silver compounds. To address these challenges, in this study we developed a process for the antibacterial modification of polyurethane foam dressings using hyperbranched polylysine. The modification process consisted of a simple five-step procedure (soaking–drying–reacting–washing–drying) that did not compromise the foam’s intrinsic properties, including hydrophilicity, pore structure and mechanical performance. The optimized modified foam achieved above 99.9% killing of both methicillin-resistant *Staphylococcus aureus* and *Escherichia coli* under experimental conditions, competitive with the commercial silver-containing dressing. The HBPL-modified foam dressings also exhibited Grade 1 cytotoxicity and a hemolysis ratio below 1%, showing good biosafety far better than that of silver dressings. In an infected burn wound model *in vivo*, the modified foam dressings achieved complete wound closure within 21 d, similar to that of silver dressing. More importantly, the healing quality was significantly improved, including enhanced inflammation resolution, angiogenesis and collagen remodeling. Given the convenience of operation and the success of modification, the method to prepare antibacterial foam dressings proposed here has great potential for applications in real world.

## Graphical abstract

**Graphical Abstract:**
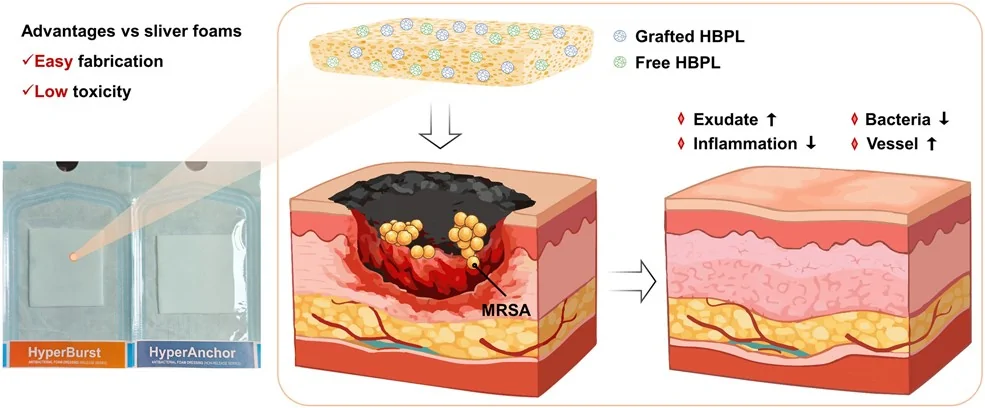

## Introduction

Foam dressings, characterized by their interconnected open-cell architecture [1], represent a cornerstone in modern wound management due to their exceptional exudate handling capacity. By absorbing fluids equivalent to dozens of times their dry weight [2, 3], these dressings maintain an optimal wound microenvironment and protect the periwound skin from maceration [4]. Moreover, foam dressings have inherent flexibility and mechanical cushioning properties [5], and are extensively utilized in the treatment of pressure injuries [6], venous ulcers [7] and diabetic ulcers [8]. More importantly, against the backdrop of increasing attention paid to multiple or drug-resistant infections, antibacterial foam dressings have emerged as the preferred choice for managing infected, high-exudate wounds such as deep burns [9] in the current medical device market. To date, commercial clinical success is almost exclusively dominated by silver-based formulations by utilizing agents such as silver nanoparticles (AgNPs), silver sulfadiazine, silver sodium zirconium phosphate, silver sulfate and silver chloride [10]. Representative commercial products include Mepilex Ag (Mölnlycke Health Care) [11], Allevyn Ag (Smith and Nephew) [12], Biatain Ag (Coloplast) [13] and so on. According to statistics, the global silver-related dressing market size reached 1.03 billion USD in 2024, and is expected to reach 1.36 billion USD by 2030 [14]. These products achieve rapid bactericidal effects by releasing silver ions (Ag^+^) into the wound bed, a mechanism that has been clinically validated for decades as highly effective in reducing bacterial bioburden [15].

Despite their commercial success, the inherent toxicity of silver-containing medical devices has become an unavoidable concern for clinicians and regulatory bodies. Ag^+^ exhibits non-selective cytotoxicity, leading to irreversible oxidative damage to healthy fibroblasts and keratinocytes while killing pathogens [16]. More critically, recent toxicological evidence highlights the risk of systemic accumulation [17]. Silver can persist in the skin, liver, kidneys and other organs or even traverse the blood–brain barrier, potentially leading to long-term neurological impairment [18]. Studies have shown that the serum silver content of patients who have used silver dressings can exceed 200 μg/L, which is more than 200 times of the normal concentration [19]. Consequently, major regulatory agencies have tightened oversight on silver-based dressings [20, 21], establishing a clear clinical demand for a transition toward non-silver, highly biocompatible antibacterial alternatives that maintain commercial-grade efficacy.

In response to these challenges, various non-silver strategies have been explored [22], including the incorporation of other metallic nanoparticles (gold [23] and ZnO [24]) or natural antimicrobial agents (chitosan [25], ε-polylysine [26] and plant extracts [27–29]). Antimicrobial coatings with mangosteen extract [30] and zinc-containing carbon dots [31] have also been studied. Because the most commonly used raw material for foam dressings is foamed polyurethane (PU) [32], these approaches often suffer from fundamental engineering limitations that hinder their industrial translation. Most existing methods incorporate the antibacterial components directly during the foaming stage. This often disrupts the delicate foaming chemistry, leading to compromised mechanical properties and poor batch-to-batch consistency. Any minor adjustment to the antibacterial load requires a complete recalibration of the raw material formulation, resulting in a prolonged development cycle and low versatility. Alternatively, simple physical adsorption methods lead to unstable coatings that lack long-term efficacy and fail to confer intrinsic antibacterial properties to the PU matrix. In contrast, chemical modification offers a viable route to overcome these drawbacks. By decoupling the synthesis of the foam matrix from its functionalization, the two processes exert minimal mutual interference, facilitating the independent optimization of their respective parameters. Furthermore, the chemical bonding ensures that the antibacterial moieties are firmly immobilized on the foam, providing sustained antimicrobial efficacy. Hyperbranched polylysine (HBPL) presents a compelling candidate for this purpose. Characterized by a high density of primary amine groups and a unique topological structure, HBPL offers exceptional antimicrobial efficiency and superior biosafety [33–35]. Drawing inspiration from PU chemical recycling paradigms [36], we propose herein the utilization of the abundant amine groups of HBPL to initiate a controlled, limited aminolysis of the pre-formed PU foam matrix. This strategy allows the foam substrate to be manufactured using mature, optimized industrial processes, with antibacterial properties added via a post-treatment technical route.

In the present study, a novel strategy for the antibacterial modification of PU foam using HBPL is developed, and the processing parameters are systematically optimized (Graphical abstract). This approach effectively circumvents the reliance on silver-based antimicrobial agents while eliminating the need for re-adjusting the foaming formulation. The modified foam dressings, which integrate both grafted and free HBPL, are employed to treat methicillin-resistant *Staphylococcus aureus* (MRSA) infected deep burn wounds, achieving therapeutic outcomes that surpass those of the traditional silver-containing dressings. It is expectable that the HBPL-modified foam dressings possess substantial translational potential and promising commercialization prospects.

## Materials and methods

### Materials

HBPL (M_n_ 5418, PDI = 1.53) was provided by Zhejiang Bioway Biotechnology Co., Ltd., China. Medical-grade PU soft foam rolls (3 mm thickness) were purchased from Nantong Dagong Haimian Co., Ltd. (Nantong, China). The commercial silver-containing foam dressing (Mepilex Ag, utilizing Ag_2_SO_4_ as the antimicrobial agent) was purchased from Mölnlycke Health Care (Gothenburg, Sweden). Medical-grade thermoplastic polyurethane (TPU) pellets (grades 1175 and 1506) were purchased from BASF (Ludwigshafen, Germany). Ninhydrin and sulfo-Cy7-NHS ester were purchased from Shanghai Aladdin Biochemical Technology Co., Ltd. (Shanghai, China). Agar, isopropanol and D201 anion exchange resin were purchased from Shanghai Macklin Biochemical Co., Ltd. (Shanghai, China). Ethanol was purchased from Sinopharm Chemical Reagent Co., Ltd. (Shanghai, China). Luria-Bertani broth powder, phosphate-buffered saline (PBS), trypsin, 3-(4,5-dimethylthiazol-2-yl)-2,5-diphenyltetrazolium bromide (MTT), Calcein/PI cell viability/cytotoxicity assay kit, reactive oxygen species (ROS) assay kit, mitochondrial membrane potential (ΔΨm) assay kit (JC-1) and paraformaldehyde (PFA) were purchased from Beyotime Biotechnology (Shanghai, China). Isoflurane was purchased from Beijing Solarbio Science & Technology Co., Ltd. (Beijing, China). Dulbecco’s modified Eagle medium (DMEM; supplemented with 100 μg/mL streptomycin and 100 U/mL penicillin) and fetal bovine serum (FBS) were purchased from Thermo Fisher Scientific (MA, USA). Anticoagulated sheep blood was purchased from Biocman Biotechnology Co., Ltd. (Changde, China). All enzyme-linked immunosorbent assay (ELISA) kits were purchased from Boster Biological Technology (CA, USA). Antibodies against Collagen I, Collagen III, cluster of differentiation 31 (CD31), alpha-smooth muscle actin (α-SMA), inducible nitric oxide synthase (iNOS) and cluster of differentiation 206 (CD206) were purchased from Abcam (Cambridge, UK).

### Preparation process of antibacterial foam dressings

#### Modification steps

The preparation of the antibacterial foam dressings followed a five-step procedure: soaking, drying, reacting, washing and drying. As a typical example, the medical-grade PU soft foam (3 mm thickness) was cut into specific dimensions (e.g., 10 cm × 10 cm or 2 cm × 2 cm), and immersed in an excess volume of HBPL solution for 30 min at room temperature. The HBPL loading capacity was modulated by adjusting the concentration of the HBPL solution. The saturated foam was then transferred to a vacuum microwave drying oven (JBL-BMV-1000D, Jinbaili, China), and dried under conditions of −0.095 MPa and 600 W for 35 min. The temperature was strictly maintained below 50°C throughout the drying process. For samples with a total surface area exceeding 300 cm^2^ (calculated in the dry state), the drying duration was appropriately extended or the samples were processed in batches. The dried foam was then subjected to a thermal reaction in a conventional oven. Following the reaction, the foam dressings containing both grafted and free HBPL were obtained directly without further processing (HyperBurst (HB) Series). In addition, to obtain the dressings containing only grafted HBPL (HyperAnchor (HA) Series), the samples were washed repeatedly with deionized water (4–5 times), and dried thoroughly in an oven at 60°C.

#### Reusability assessment of HBPL solutions

HBPL solutions (120 mL each) were prepared at concentrations of 1, 2.5 and 5 mg/mL. For each concentration, 0.5 g of foam was immersed in the solution for 30 min. After removal of the foam, a micro-volume aliquot of the solution was collected for subsequent analysis. This immersion-sampling procedure was repeated for 10 consecutive cycles. The variations in HBPL concentration throughout the repetitive process were determined using the ninhydrin assay [37].

#### Determination of amino group content via ninhydrin test

The reaction mixture was prepared by adding 1 mL of 0.1 M ethanolic ninhydrin solution to either 0.1 mL of the liquid sample or 15 mg of the solid sample. The reaction was allowed to proceed at 60°C for 15–30 min. Subsequently, the absorbance at 570 nm was recorded using a microplate reader (Infinite 200 Pro, Tecan, Switzerland).

#### Comparison of drying methods

The foam samples (10 cm × 10 cm) were immersed in a 1 mg/mL HBPL solution for 30 min, followed by drying via three distinct techniques: microwave irradiation, hot air convection and infrared radiation. The microwave drying parameters were consistent with those previously specified. Both the hot air and infrared drying were conducted at 120°C for 2 h. The total content of HBPL in the foams obtained by each method was tested by the ninhydrin method. Each dried foam was uniformly partitioned into nine equal segments. To evaluate the spatial distribution uniformity of HBPL within the foam matrix, each segment was individually extracted in 20 mL of PBS at 37°C for 12 h. The HBPL concentration in the resulting extracts was quantified using the ninhydrin assay.

### Release of HBPL *in vitro*

To evaluate the release behavior of the antibacterial foam dressings obtained without post-reaction washing, samples were prepared using various HBPL concentrations (1, 2.5 and 5 mg/mL) and subjected to different reaction times (0, 5, 15, 35 and 65 min). Each foam sample (0.5 g) was incubated in 20 mL of PBS at 37°C for 12 h to ensure complete extraction. The amount of HBPL released into the supernatant was quantified using the ninhydrin assay. The results were normalized to the initial dry weight of the foams to determine the HBPL release per unit mass. Furthermore, for the samples with reaction duration of 0 and 5 min, aliquots of the supernatant were collected at predetermined time intervals, with an equal volume of fresh PBS replenished to maintain a constant total volume. The concentration of HBPL released at each time point was quantified using the ninhydrin assay, and normalized to the dry weight of the foams.

### Chemical structure characterization of modified foams

The loading and grafting of HBPL onto the foam were characterized using a Fourier transform infrared spectrometer (FTIR; Tensor II, Bruker, Germany) equipped with an attenuated total reflection accessory. Spectra were acquired over a scanning range of 4000–400 cm^−1^ with a spectral resolution of 0.4 cm^−1^, accumulating 32 scans per sample. Prior to each measurement, the crystal surface was cleaned with ethanol, and an ambient air background was collected for subtraction. Samples were placed directly onto the crystal and compressed to ensure intimate contact.

X-ray photoelectron spectroscopy (XPS; Axis Supra+, Kratos, Japan) was employed to determine the surface nitrogen (N 1s) content. Monochromatic Al Kα radiation (hν = 1486.6 eV) was used as the excitation source. High-resolution N 1s spectra were collected at 20 eV pass energy. All spectra were calibrated using the adventitious carbon (C 1s) peak at 284.8 eV as an internal reference.

### Universal testing of reaction principles

TPU particles, including both polyether-based type (1175) and polyester-based type (1506), were used to simulate the grafting reaction. For each type, 0.5 g of TPU particles were placed in a 6-well plate and submerged in 2 mL of a 5 mg/mL HBPL solution. The samples were initially heated at 60°C to evaporate the solvent, followed by a thermal treatment at 150°C for 30 min to initiate the reaction. After the reaction, the particles were thoroughly washed to remove any non-bonded HBPL. The density of the grafted amino groups on the particle surfaces was subsequently quantified using the ninhydrin assay.

### Characterization of the basic properties of foams

#### Microstructure

The surface morphology and pore structure of the foam samples were observed using a field-emission scanning electron microscope (SEM; SU8600, Hitachi, Japan). Prior to imaging, the samples were subjected to vacuum gold sputtering for 30 s to enhance their electrical conductivity. All micrographs were acquired at an accelerating voltage of 3 kV.

#### Porosity

Foam samples were immersed in an excess of light liquid paraffin. To ensure full saturation, the samples were ***centrifuged*** at 2500 rpm for 5 min, and the volume was recorded. The foams were then removed from the paraffin, and the volume was recorded again. The porosity was calculated according to the following equation:


$$\mathrm{Porosity (\%)}=\frac{V_{1}-V_{3}}{V_{2}-V_{3}}\times 100\%,$$

where *V*_1_, *V*_2_ and *V*_3_ represent the volumes of liquid paraffin before the foams were added, after the foams were completely saturated and after the foams were removed, respectively.

#### Absorption ratio

Foam samples were immersed in excess PBS for 5 min, hung to allow the removal of excess water and then weighed. The absorption ratio was calculated according to the following formula:


$$\mathrm{Absorption}\;\mathrm{ratio}=(\frac{m_{\mathrm{wet}}}{m_{\mathrm{dry}}}-1)\times 100\%,$$

where *m*_dry_ and *m*_wet_ represent the mass of the foams before and after water absorption, respectively.

#### Instantaneous water absorption

The instantaneous water absorption behavior of the foam samples was characterized by using a contact angle goniometer (DSA100, Krüss, Germany). A 2-μL droplet of deionized water was deposited onto the foam surface, and the static water absorption image was captured after a 2 s equilibration period.

#### Mechanical properties

The mechanical performance of the foams was evaluated using a universal testing machine (5543A, Instron, USA). For compression tests, the foam samples were cut into rectangular blocks with dimensions of 5 mm × 5 mm × 3 mm. The compression was performed at a constant rate of 2 mm/min over a strain range of 0–70%. For the tensile tests, rectangular specimens (50 mm × 10 mm × 3 mm) were tested at a speed of 20 mm/min to determine the ultimate tensile strength and elongation. The elastic modulus (Young’s modulus) was calculated as the slope of the linear portion of the stress–strain curve within the 20–30% strain range.

### Antibacterial performance tests *in vitro*

The antibacterial efficacy of the foam dressings was evaluated using two complementary methods: the contact assay and the release assay. For contact assay, the foam samples (8 mm × 8 mm) were sterilized by ultraviolet irradiation for 0.5 h on each side. The samples were placed in culture dishes, and 100 μL of an MRSA (CICC 25138, China Center of Industrial Culture Collection) or *Escherichia coli* (*E. coli*; CICC 10305, China Center of Industrial Culture Collection) bacterial suspension (10^6^ CFU/mL) was inoculated directly onto the foam surface. Following incubation at 37°C for 12 h, the foams were transferred into 5 mL of PBS and subjected to ultrasonication for 5 min to detach the adhered bacteria. Subsequently, 50 μL of the resulting bacterial suspension was spread onto agar plates and incubated at 37°C for another 24 h. The limit of detection (no colonies on the plate) in this method is 20 CFU/mL. For release assay, the foam specimens (25 mg) were immersed in 2 mL of an MRSA or *E. coli* bacterial suspension (10^6^ CFU/mL), and incubated in a shaking incubator at 37°C for 12 h. The post-incubation suspension was diluted 10-fold, and 50 μL was spread onto agar plates. After incubation at 37°C for 24 h, the plates were photographed, and the colony numbers were quantified. The limit of detection in this method is 200 CFU/mL. In both assays, unmodified foams served as the control group. The antibacterial rate was calculated using the following equation:


$$\mathrm{Antibacterial}\;\mathrm{rate}\;(\%)=(1-\frac{n_{\mathrm{sample}}}{n_{\mathrm{control}}})\times 100\%,$$

where *n*_sample_ and *n*_control_ represent the colony numbers of the experimental group and the control group, respectively.

### Biocompatibility tests

#### Cell compatibility tests

L929 mouse fibroblasts (GNM28, National Collection of Authenticated Cell Cultures, Chinese Academy of Sciences) were employed for the biocompatibility tests. The culture medium consisted of 90% (v/v) high-glucose DMEM supplemented with 10% (v/v) FBS. To prepare the extracts, foam samples were immersed in DMEM at a liquid-to-mass ratio of 24 mL/g and incubated at 37°C for 3 d. The foams were then removed, and the resulting extract was supplemented with FBS for subsequent use. For the cytotoxicity tests, cells were seeded in 96-well plates at a density of 8 × 10^3^ cells per well, and incubated in a humidified atmosphere with 5% CO_2_ at 37°C for 1 d. Subsequently, the culture medium was replaced with the prepared extract, and the cells were cultured for predetermined time.

After incubation with the extract for 1 d or 3 d, the culture medium was replaced with a fresh medium containing 1 mg/mL MTT. Following a 2 h incubation period to allow for the formation of formazan crystals, the medium was removed and replaced with isopropanol to dissolve the crystals. The absorbance of the resulting supernatant was measured at 570 nm using a microplate reader. Cells cultured in standard medium served as the positive control group, while the group without cells but only reagents served as the blank control group. The cell viability was calculated according to the following equation:


$$\mathrm{Cell}\;\mathrm{viability}\;(\%)=\frac{{\mathrm{OD}}_{\mathrm{sample}}-{\mathrm{OD}}_{\mathrm{blank}}}{{\mathrm{OD}}_{\mathrm{positive}}-{\mathrm{OD}}_{\mathrm{blank}}}\times 100\%,$$

where OD_sample_, OD_blank_ and OD_positive_ represent the absorbance readings of the experimental group, the blank control group and the positive control group, respectively.

In addition, the effect of filtration of Mepilex Ag extracts on L929 cell viability after 1 day of incubation was evaluated using the same method described above.

A live/dead dual-staining assay was also performed after the fibroblasts were incubated with the extract for 1 d or 3 d. A staining solution containing 2× Calcein-AM and 1× propidium iodide (PI) was then added, and the cells were further incubated in a CO_2_ incubator for 30 min. Following a thorough rinsing step to remove excess fluorophores, the cell morphology and viability were visualized using an inverted fluorescence microscope (IX81, Olympus, Japan).

#### Hemolysis test

Anticoagulated sheep blood was employed to evaluate the hemocompatibility of the foam dressings. A diluted blood suspension was prepared by mixing the anticoagulated sheep blood with PBS at a volume ratio of 1:7. Subsequently, 10 mg of the foam sample was immersed in 1 mL of the diluted blood and incubated at 37°C for 1 h. Following incubation, the mixture was subjected to centrifugation at 800 g for 5 min. The absorbance of the resulting supernatant was measured at 545 nm using a microplate reader to quantify the released hemoglobin. Diluted blood prepared using deionized water served as the positive control group. The hemolysis ratio was calculated according to the following equation:


$$\mathrm{Hemolysis}\;\mathrm{ratio}\;(\%)=\frac{{\mathrm{OD}}_{\mathrm{sample}}-{\mathrm{OD}}_{\mathrm{blank}}}{{\mathrm{OD}}_{\mathrm{positive}}-{\mathrm{OD}}_{\mathrm{blank}}}\times 100\%,$$

where OD_sample_, OD_blank_ and OD_positive_ represent the absorbance readings of the experimental group, the blank group and the positive control group, respectively.

#### Subcutaneous implantation test

All animal procedures were approved by the Lab of Animal Experimental Inspection of Dr Can Biotechnology (Zhejiang) Co., Ltd. (Reference number: DRK-202511073648). Eight-week-old C57BL/6 mice (SPF grade, Vital River) were anesthetized by inhalation with 2% isoflurane for 5 min. A 1-cm longitudinal incision was made on one side of the mouse’s back, and a subcutaneous pocket was created through blunt dissection. One foam dressing sample (15 mg) was implanted into the pocket, followed by wound closure with sutures. After 30 d, the mice were euthanized to harvest the samples. Blood samples were collected for hematological and biochemical analysis using an automated hematology analyzer (XN-1000V, Sysmex, Japan) and an automated chemistry analyzer (Cobas c501, Roche, Switzerland), respectively. Major organs, the implanted dressings and the surrounding tissues were excised and fixed in 4% PFA. These samples were subsequently embedded in paraffin and sectioned. Hematoxylin and eosin (H&E) staining was performed on the sections, and the inflammatory response at the implantation site was evaluated using a slide scanner (VS200, Olympus, Japan).

### Mechanisms of the differences in biological safety

#### Comparative cytotoxicity tests

Building upon the previously described biocompatibility protocols, the extraction ratio was adjusted to 80 mL of DMEM per 1 g of foams. The extract from the commercial Mepilex Ag dressing was further diluted to various concentrations to evaluate its dose-dependent toxicity. Additionally, a short-term exposure model was established by reducing the incubation time with the extracts to 20 min to assess immediate cytotoxic effects.

#### Intracellular ROS staining

Following a 20 min stimulation with the foam extracts, the medium was replaced with a staining solution containing 10 μmol/L of a probe (DCFH-DA). The cells were incubated for 20 min in a CO_2_ incubator. After thoroughly washing, the intracellular fluorescence intensity was observed to evaluate oxidative stress levels.

#### ΔΨm staining

After 20 min of stimulation with the extracts, the cells were incubated with a 1× JC-1 staining working solution for 20 min. Following the washing steps, the shifts in fluorescence (from red aggregates to green monomers) were observed to assess mitochondrial depolarization.

#### Flow cytometry analysis of live/dead staining

Cells were seeded in 6-well plates at a density of 2 × 10^5^ cells per well. After 24 h of incubation, the cells were stimulated with the extracts for 20 min. Live/dead staining was then performed according to the protocols described in Section 2.8. In addition to microscopic observation, the cells were trypsinized, harvested and analysed using a flow cytometer (FACSCalibur, BD, USA) to quantify the fluorescence distribution.

### Long-term storage and sterilization stability

HB1 and HA5 were placed in self-sealing sterilization pouches and either stored at room temperature for 300 days or sterilized by electron-beam irradiation (15 kGy; CIAE-FZ-10/15). Their antibacterial activity was subsequently evaluated according to the methods described in Section 2.7, while the total amino group content and amount of releasable amino groups were determined according to the methods described in Sections 2.2 and 2.3.

### Wound healing evaluation in an MRSA-infected deep burn model

Eight-week-old C57BL/6 mice were anesthetized by inhalation with 2% isoflurane for 5 min before their dorsal hair was removed using a hair clipper and depilatory cream. To create a third-degree burn wound, a brass rod (80 g, 10 mm diameter), preheated in a boiling water bath for 10 min, was applied vertically to the dorsal skin for 30 s, ensuring that the pressure was exerted solely by the gravity of the rod. 6 h post-burn, the necrotic skin was excised along the wound margins to simulate clinical debridement. Subsequently, 40 μL of an MRSA suspension (10^6^ CFU/mL) was inoculated onto the wound site to establish the infection. Treatment was initiated 24 h after bacterial inoculation by applying the foam dressings, which were secured with a transparent film (3M Tegaderm Film, 3M Health Care, USA). The dressings were changed every 3 days, and the wounds were photographed to monitor the healing process until complete re-epithelialization. The wound healing rate was calculated using the following equation:


$$\mathrm{Wound}\;\mathrm{healing}\;\mathrm{rate}\;(\%)=(1-\frac{S_{t}}{S_{0}})\times 100\%,$$

where *S*_t_ and *S*_0_ represent the wound areas on day *t* and day 0, respectively.

A subset of mice was euthanized 6 d post-treatment to harvest wound tissues. The tissues were homogenized to quantify the bacterial burden and the levels of inflammatory cytokines. Upon complete wound closure, the remaining mice were euthanized, and the regenerated wound tissues were collected and fixed in 4% PFA. Following paraffin embedding and sectioning, the sections were subjected to H&E staining and Masson’s trichrome staining. Furthermore, immunofluorescence staining was performed, including type I and type III collagen staining, CD31 and α-SMA staining, and iNOS and CD206 staining. After the sections were blocked with 5% BSA for 1 h, they were incubated overnight at 4°C with primary antibodies. After washing, the sections were incubated with corresponding fluorophore-conjugated secondary antibodies for 1 h at room temperature in the dark. Nuclei were counterstained with DAPI. All stained sections were visualized using a slide scanner to evaluate the overall quality of wound healing and tissue regeneration.

### Release of HBPL *in vivo*

HBPL was conjugated with sulfo-Cy7-NHS ester for visualization. Briefly, 1 g of HBPL was dissolved in 100 mL of deionized water, whose pH value was adjusted to 8.5. After 2.3 mg of sulfo-Cy7-NHS ester was added, the mixture was allowed to react for 24 h at room temperature under stirring in the dark. The resulting solution was diluted 10-fold before it was purified by D201 anion exchange resin to remove unreacted sulfo-Cy7-NHS ester. The purified solution was then lyophilized to obtain the fluorescently labeled HBPL.

The antibacterial foam dressings were then prepared by using the fluorescently labeled HBPL, which were then applied to the surface of fresh excisional wounds in mice. After treatment for 3 d, the dressings were removed, and the wound site was imaged using an *in vivo* imaging system to detect the residual fluorescence intensity and the spatial distribution of HBPL within the wound bed.

### Statistical analysis

All experimental data are presented as means ± SD. Statistical significance between groups was determined using one-way analysis of variance (ANOVA). Data processing and visualization were performed using GraphPad Prism software (GraphPad Software Inc., USA). A *P*-value of less than 0.05 was considered statistically significant. The level of significance is indicated as follows: *P < 0.05, **P < 0.01, ***P < 0.001 and ****P < 0.0001.

## Results and discussion

### Modification process

In contrast to conventional methodologies that incorporate antimicrobial agents during the initial polymerization of foam dressings, a post-functionalization approach was applied to the pre-synthesized foams. This strategy bypasses the need to re-optimize the complex synthesis formulations and manufacturing parameters, thereby ensuring maximum compatibility with the existing commercial foam production lines. The specific modification protocol is streamlined into five sequential stages: soaking, drying, reacting, washing and final drying. These steps are designed to achieve HBPL loading, moisture removal, covalent immobilization of HBPL, elimination of unbound HBPL and final dehydration, respectively. Subsequently, key processing parameters were evaluated and optimized.

As illustrated in Figure 1A, Step 1 involves the thorough immersion of unmodified foams in an excess of HBPL solution. First, the reusability of the HBPL solution was evaluated. As shown in Figure 1B, the absorbance values of the three solutions at varying concentrations remained remarkably stable throughout the 10 consecutive immersion cycles, suggesting the constant HBPL concentrations and thereby reusability of HBPL solutions and batch stability of the final products. This phenomenon can be only explained by the physical entrapment of HBPL solution without any specific attraction and adsorption of HBPL molecules onto the PU foams.

**Figure 1:**
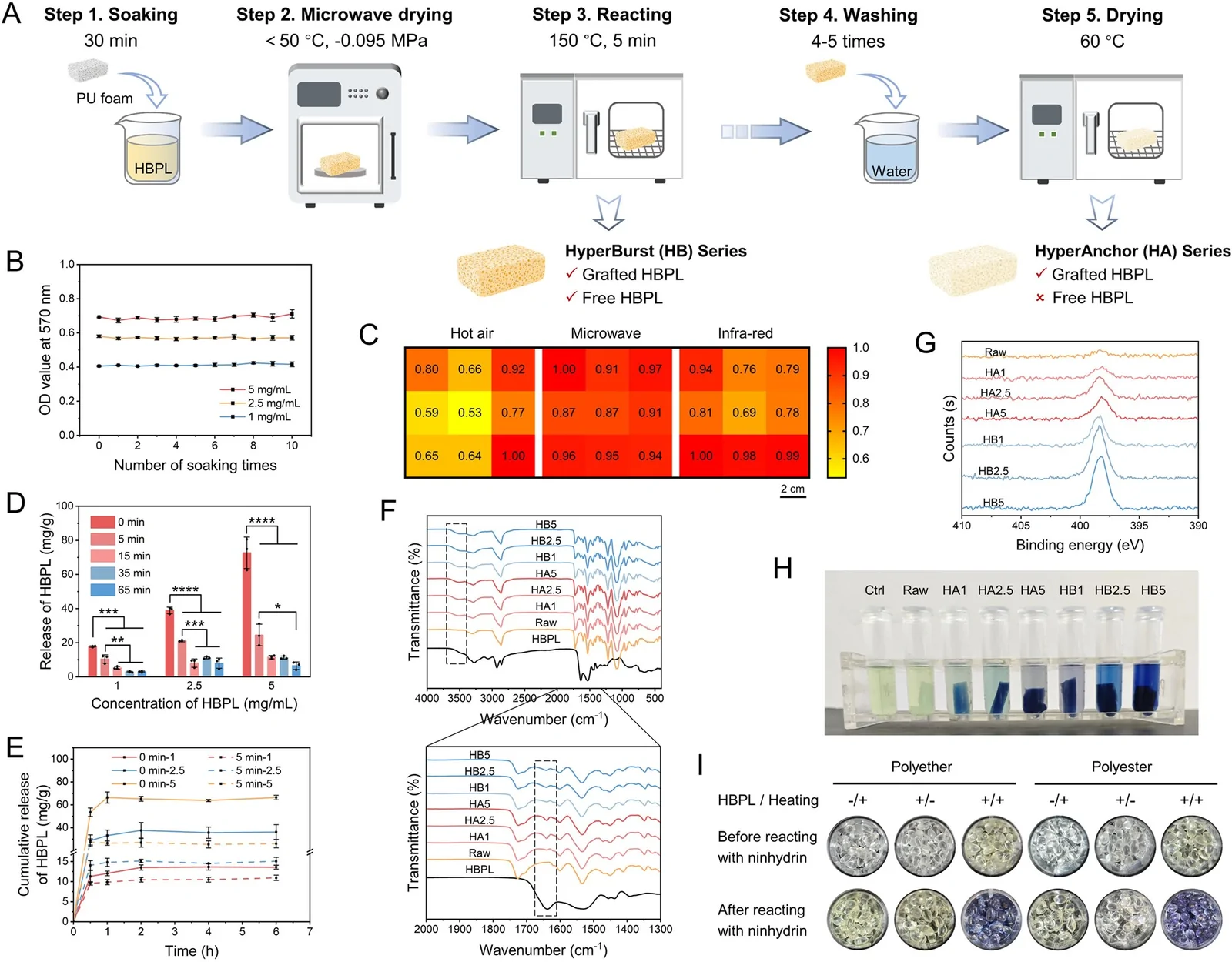
Modification processes and characterizations of foam dressings by HBPL. (**A**) Schematic diagram of the modification processes with HBPL to obtain the antibacterial foam dressings without (HyperAnchor series, washed) and with free HBPL (HyperBurst series, not washed). (**B**) Concentration variation of HBPL solution during repeated incubation (*n* = 3). (**C**) Uniformity of HBPL distribution under different drying methods. (**D**) Total release and (**E**) release curve of HBPL under different reaction time and concentrations (*n* = 3). (**F**) FTIR and (**G**) XPS spectra of modified foams under different conditions. Color development photos of HBPL-modified (**H**) foam and (**I**) TPU particles using ninhydrin assay. Data are presented as means ± SD. *, **, *** and **** represent *P* < 0.05, 0.01, 0.001 and 0.0001, respectively.

Step 2 involves drying of the fully saturated foams to retain the HBPL for subsequent chemical reactions. In this stage, three distinct drying methodologies of microwave, infrared and hot air drying were compared to assess their impact on the spatial distribution uniformity of the loaded HBPL. The total content of HBPL in the foams obtained by the three drying methods was consistent without apparent difference (Figure S1). The dried samples were then partitioned into nine equal segments to quantify the relative HBPL content in each segment. Figure 1C demonstrates that the microwave drying achieved the best homogeneous distribution compared with the other methods. This performance is likely attributable to the enhanced efficiency of microwave drying, which requires only 0.5 h for complete dehydration. It was 1.5 h faster than the infrared or hot air drying, thereby significantly minimizing the relocation of HBPL solutions during the evaporation process. Consequently, microwave drying was selected as the standard protocol for Step 2 in all subsequent experiments.

Step 3 involves the thermal treatment of the HBPL-loaded foams at 150°C to chemically anchor a portion of the HBPL onto the foam. We investigated the extent of the grafting reaction by modulating the reaction time and the initial HBPL concentration. As shown in Figure 1D, at a constant concentration, prolongation of the reaction time led to a reduction in the amount of released HBPL, indicating an increase in the grafted fraction. Notably, ∼50% of the loaded HBPL was successfully grafted within just 5 min of reaction. The immobilization efficiency reached a plateau beyond 30 min, showing no further significant increase. To clearly differentiate between the effects of grafted and free HBPL, a reaction time of 5 min was standardized for all subsequent experiments. Meanwhile, increasing the initial HBPL concentration (from 1 to 5 mg/mL) resulted in a higher total amount of released HBPL. The cumulative release profiles (Figure 1E) further confirm that the samples reacted for 5 min (denoted as 5 min-1, 5 min-2.5 and 5 min-5) exhibited significantly lower HBPL release compared to their unreacted counterparts (0 min-1, 0 min-2.5 and 0 min-5). Regarding the release kinetics, HBPL liberation was remarkably rapid, reaching equilibrium within 0.5–1 h. This rapid release behavior is comparable to the release rate of Ag^+^ from Mepilex Ag (Figure S2), ensuring a swift antimicrobial response upon application.

Steps 4 and 5 involve the repeated manual squeezing and rinsing of the foams with deionized water to eliminate any unreacted HBPL, followed by final dehydration. Since these stages no longer involve the relocation of HBPL, a standard hot air convection oven was employed for drying. The nomenclature for the samples used in the subsequent experiments is summarized in Table 1. The pristine PU foam without any modification is designated as Raw. For samples that underwent the complete five-step process, where HBPL exists only in a covalently grafted state, they are named as the HyperAnchor (HA) series (e.g., HA1, HA2.5 and HA5). Conversely, samples prepared by omitting Steps 4 and 5, which contain both free and grafted HBPL, are denoted as the HyperBurst (HB) series (e.g., HB1, HB2.5 and HB5). A commercially available silver-containing foam dressing Mepilex Ag (incorporated with Ag_2_SO_4_) was selected as a commercial benchmark.

**Table 1:** The grouping of materials in this study.

Group	Modification operation	Antibacterial agent
Raw	None	None
HA1	Steps 1–5	Grafted HBPL
HA2.5	Steps 1–5	Grafted HBPL
HA5	Steps 1–5	Grafted HBPL
HB1	Steps 1–3	Grafted and free HBPL
HB2.5	Steps 1–3	Grafted and free HBPL
HB5	Steps 1–3	Grafted and free HBPL
Mepilex Ag	None	Free Ag_2_SO_4_

The evolution of chemical functional groups following HBPL modification was monitored via FTIR spectroscopy. As shown in Figure 1F, the spectra of the modified foams exhibit two prominent changes compared to the Raw group: a significantly enhanced peak at ∼1639 cm^−1^, corresponding to the C = O stretching vibration, and a broadened absorption band around 3500 cm^−1^, attributed to N–H stretching vibrations. These features are characteristic of the amide and amino groups inherent to HBPL. We propose that the mechanism of modification involves a nucleophilic substitution reaction between the amino groups of HBPL and the urethane linkages of the PU foam matrix at elevated temperatures, leading to the formation of stable urea bonds [38, 39]. This reaction mechanism is also supported by the increased intensity of the carbonyl peak at 1639 cm^−1^. Surface elemental composition was further analysed using XPS. As shown in Figure 1G, the nitrogen (N 1 s) content increased significantly in the samples processed through Steps 1–3 (HB1, HB2.5 and HB5). Although the nitrogen signal intensity decreased after the washing process (HA1, HA2.5 and HA5), it remained substantially higher than that of the Raw group, and showed a positive correlation with the initial HBPL concentration. Furthermore, the presence of surface amino groups was qualitatively verified using the ninhydrin assay. As shown in Figure 1H, samples treated with higher concentrations of HBPL and those without washing exhibited a noticeably intense blue coloration, indicating a higher density of available amino groups. Collectively, these results confirm the successful incorporation of HBPL onto the foam, with the final loading amount being dependent on the initial solution concentration and the subsequent purification steps.

Furthermore, to evaluate the versatility of the proposed modification strategy, two distinct types of commercially available medical-grade TPU particles (polyester-based type and polyether-based type), were subjected to the identical modification protocol. The presence of surface amino groups was subsequently detected using the ninhydrin assay. As shown in Figure 1I, both types of TPU particles turned yellow after reaction with HBPL at 150°C, and became significantly blue after color development with ninhydrin, suggesting successful HBPL immobilization. These findings confirm the broad applicability of this modification technique across different PU substrates. Moreover, this result provides indirect evidence for the proposed reaction mechanism: the grafting occurs specifically through the reaction between the amino groups of HBPL and the urethane linkages within the PU backbone, occurring independently of the specific chemical structure of the PU soft segments.

### Influence of the modification process on foam structure and properties

Although the success of the modification has been established, it is imperative to evaluate its impact on the fundamental properties of the foam dressings. The macroscopic appearance was not affected after the incorporation of a relatively small amount of HBPL (Figure S3). Only a subtle yellowing was observed in samples treated with high HBPL concentrations (≥ 5 mg/mL), which was inherited from the yellow color HBPL. The microscopic morphology characterized by SEM (Figure 2A) showed no distinct differences before and after modification. All samples exhibited a well-distributed pore structure ranging from tens to hundreds of micrometers, with a calculated porosity of ∼80% across all groups (Figure 2B). Furthermore, the swelling ratio in PBS remained consistent at ∼1900% for all samples (Figure 2C). Instantaneous water absorption measurements confirmed that all foam variants maintained excellent hydrophilicity, capable of fully absorbing water droplets within 2 s of surface contact (Figure 2D). Since mechanical integrity is a critical factor for the practical application of foam dressings [40], both the ultimate tensile strength and compression tests (up to 70% strain) were conducted. As shown in Figure 2E and F, the mechanical profiles of all groups remained nearly identical. Specifically, the tensile strength, tensile modulus, elongation at break and stress at 70% compressive strain were distributed within the ranges of 100–120 kPa, 36–40 kPa, 450–500% and 11–14 kPa, respectively (Figure S4). These results collectively demonstrate that the HBPL-based antibacterial modification does not compromise the essential physical and mechanical attributes of the PU foams as wound dressings, suggesting the foams are stable enough through the medium and thermal treatments at the investigation conditions.

**Figure 2:**
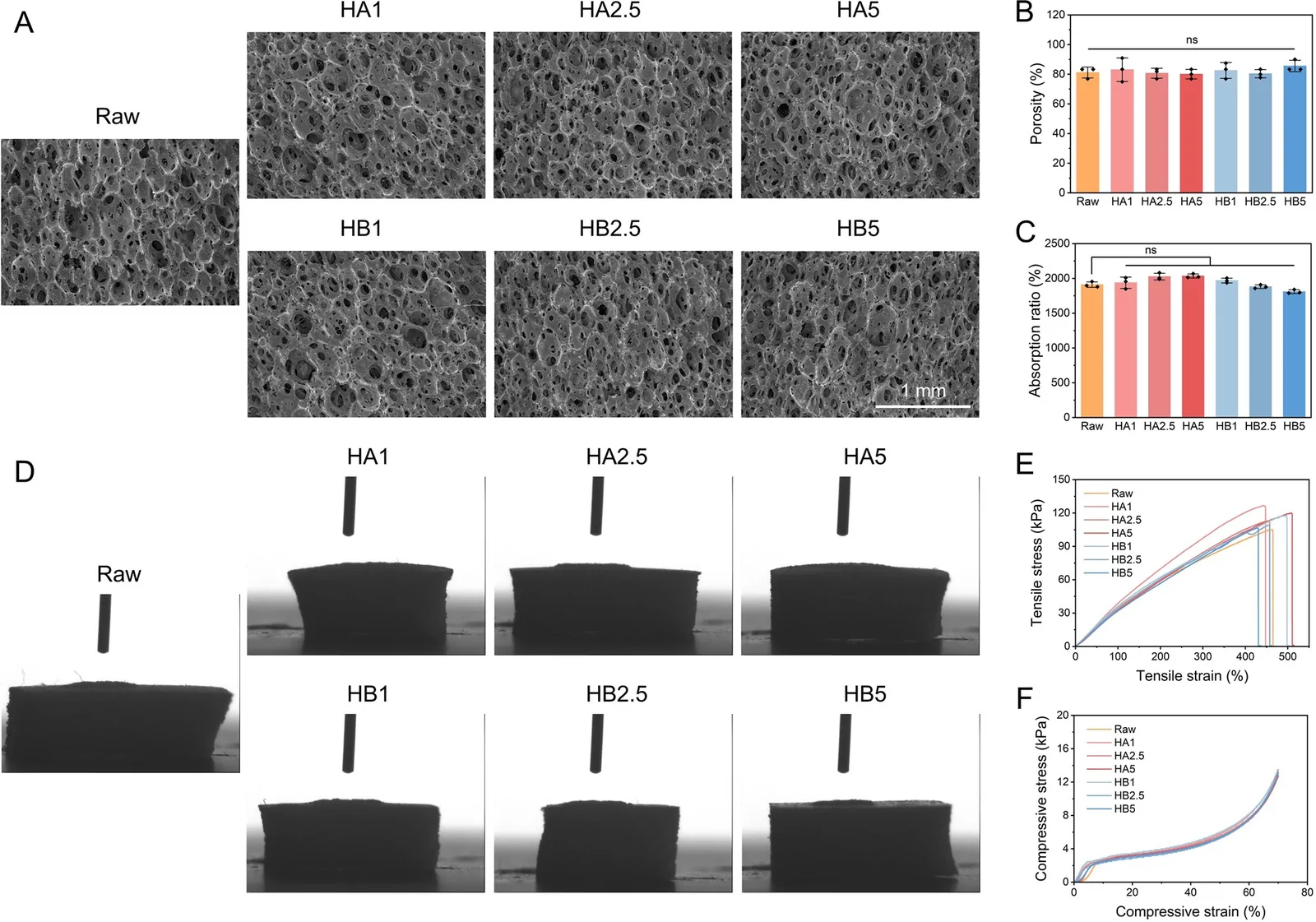
Influence of antibacterial modification on the main physiochemical properties of foam dressings. (**A**) SEM images to show their microscopic morphology. (**B**) Porosity calculated by the liquid displacement method (*n* = 3). (**C**) Liquid absorption ratio (*n* = 3). (**D**) Snapshots showing the instantaneous water absorption of foams. (**E**) Typical tensile curves, and (**F**) typical compression curves of modified foam dressings under different conditions. Data are presented as means ± SD. *ns* represents no significant difference (*P* > 0.05).

### Antibacterial performance

Endowing the foam dressings with robust antibacterial properties is the primary objective of the modification, and serves as the definitive indicator of its success. MRSA and *E. coli* were selected as representative Gram-positive and Gram-negative bacteria, respectively. The antibacterial performance of the various foams was systematically evaluated using both contact-killing and release-killing assays, and compared against the commercial benchmark (Mepilex Ag). In the contact-killing assay (Figure 3A and B), the antibacterial efficacy of the washed samples (HA1, HA2.5 and HA5) exhibited a clear concentration-dependent upward trend. Residual bacterial survival was still observed for groups HA1 and HA2.5, with only group HA5 achieving near-complete bacterial eradication. This observation is attributed to the relatively lower HBPL loading in these three groups following the rigorous washing process. In contrast, the unwashed groups (HB1, HB2.5 and HB5) demonstrated > 99.9% killing efficiency against both bacterial strains, performing at the same level as the commercial group.

**Figure 3:**
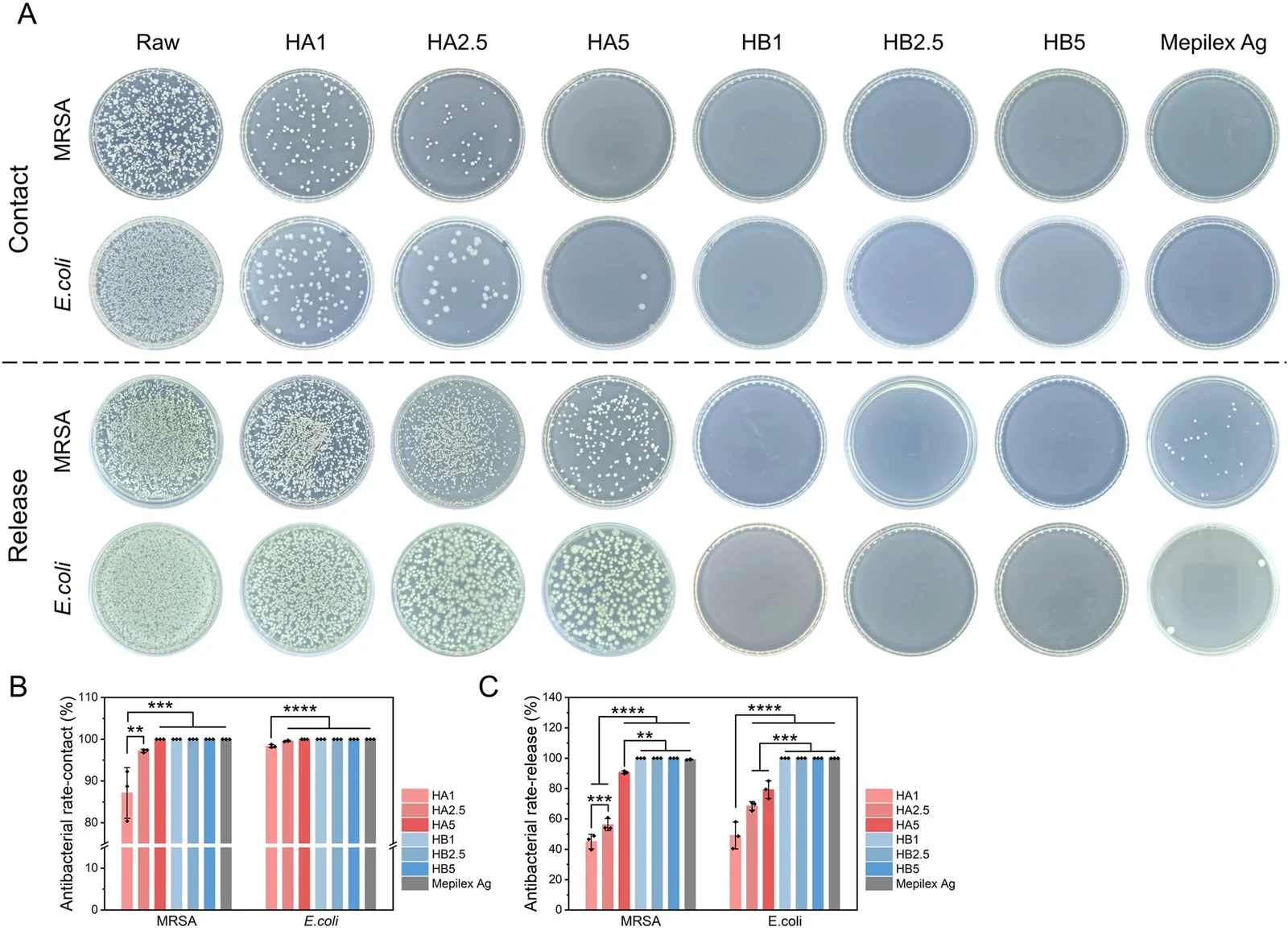
Antibacterial performance test of foam dressings. (**A**) Colony count photos of each group. For the contact method, 100 μL of MRSA or *Escherichia coli* bacterial solution (10^6^ CFU/mL) was added to the surface of a square foam with a side length of 8 mm, and incubated for 12 h. For the release method, 25 mg foam was added to 2 mL of MRSA or *Escherichia coli* bacterial solution (10^6^ CFU/mL), and incubated for 12 h. Specific antibacterial rate tested (**B**) by contact method and (**C**) by release method (*n* = 3). Data are presented as means ± SD. **, *** and **** represent *P* < 0.01, 0.001 and 0.0001, respectively.

In the release-killing assay (Figure 3A and C), the samples containing only grafted HBPL (HA1, HA2.5 and HA5) exhibited a significant decline in antibacterial efficacy. Taking group HA1 against MRSA as an example, the antibacterial rate dropped from 87% in the contact assay to 45% in the release assay. This reduction is attributed to the fact that grafted antibacterial agents can only eliminate bacteria through limited collision and surface adsorption within a stationary liquid environment. Even for the group HA5, which was treated with the highest concentration, the antibacterial rate remained below 90%. In stark contrast, the groups HB1, HB2.5 and HB5, which possessed both grafted and free HBPL, maintained an antibacterial rate close to 100%. Notably, the group Mepilex Ag showed a slight decrease in efficacy to ∼99%, potentially due to an insufficient local concentration of released Ag^+^, which was surpassed by the performance of the HB series.

These data demonstrate that the proposed modification strategy successfully achieves the objective of endowing foam dressings with potent antimicrobial activity. Furthermore, by simply modulating the modification processes, two distinct product formats (contact-active and release-active) can be easily fabricated. The release-active variants, in particular, exhibit superior antibacterial performance compared to existing commercial silver-based dressings.

### Biological safety

As medical devices, the foam dressings must strictly adhere to biocompatibility requirements. Therefore, the safety of the modified foams was systematically evaluated at the cell, blood and system levels. At the cell level, the cytocompatibility of the extracts was assessed using L929 fibroblasts through the MTT assay (Figure S5A and B) and live/dead staining (Figure 4A) over 1 d and 3 d of incubation. For the HBPL-modified groups, only the HB5 group exhibited a temporary reduction in cell viability (∼60%) after 1 d of exposure. However, its cell viability recovered to over 80% by day 3. It is speculated that, upon initial addition, HBPL electrostatically interferes with L929 adhesion, leading to transient detachment and reduced metabolic activity in some cells. As HBPL is gradually adsorbed onto the culture plate, internalized by the cells, or neutralized by proteins, this interference weakens, allowing the cells to recover their viability. All other HBPL-modified groups maintained a cell viability of above 80% throughout the 3 d culture period compared to the Ctrl group, corresponding to grade 1 cytotoxicity (with cell viability ranging from 75% to 99% [41]). Furthermore, the increase in absorbance values from day 1 to day 3 confirmed that the cells remained in a proliferative state (Figure 4B). In contrast, the Mepilex Ag group exhibited severe cytotoxicity, resulting in near-complete cell death within only 1 d of incubation. It is understandable that Ag_2_SO_4_ in Mepilex Ag combines with Cl^−^ in DMEM during extraction to generate a large amount of AgCl precipitate. To maintain consistency with the other groups, the extract was not filtered. Thus, AgCl gradually precipitated to the bottom of the well plate, almost completely covering the cells and leading to physical damage to the cells. In addition, free Ag^+^ also contributes significantly to the cytotoxicity. The data in Figure S5C show that filtration of the Mepilex Ag extract markedly reduced its effect on L929 cell viability, confirming that precipitate formation plays a dominant role in its cytotoxicity. These findings demonstrate that the adoption of HBPL as an antibacterial agent significantly enhances cytocompatibility of foam dressings compared to the silver-based alternatives. The hemocompatibility was further evaluated via the hemolysis test (Figure 4C). The hemolysis ratios for the Raw group and all HBPL-modified groups were consistently below 1%, well within the safe threshold for medical materials. Conversely, the Mepilex Ag exhibited a hemolysis ratio exceeding 20%, indicating substantial red blood cell damage.

**Figure 4:**
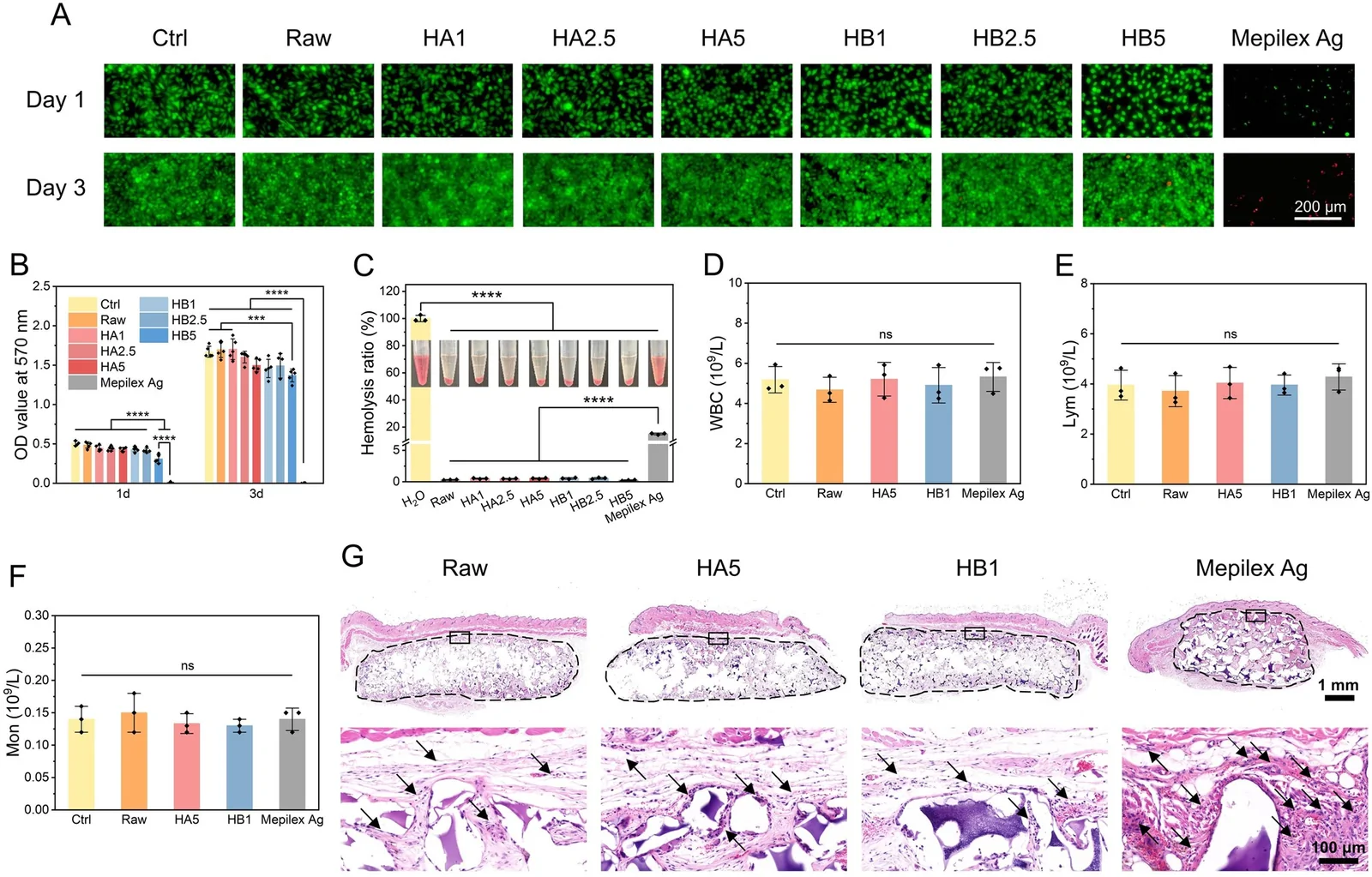
Biosafety assessment of modified foam dressings. (**A**) Live (Calcein-AM, green) and dead (PI, red) staining photos taken by fluorescence microscopy after L929 cells (8 × 10^3^ per well) were cultured with foam extracts (200 μL per well) in a 96-well plate for 1 d and 3 d, and (**B**) corresponding absorbance changes measured by the MTT method (*n* = 5). (**C**) Hemolysis ratio of each foam (*n* = 3), where insets show the corresponding photos. Numbers of (**D**) white blood cells, (**E**) lymphocytes and (**F**) monocytes in the blood measured 30 d after subcutaneous implantation (15 mg foam for each C57BL/6 mouse) of each group (*n* = 3). (**G**) H&E staining of the foams and the surrounding tissues. The dressings are outlined with the dashed lines in each figure, and the inflammatory cells are pointed out by the arrows. Data are presented as means ± SD. *** and **** represent *P* < 0.001 and 0.0001, respectively. *ns* represents no significant difference (*P* > 0.05).

Based on the aforementioned results *in vitro*, and adhering to the principle of balancing potent antibacterial activity with minimal cytotoxicity, groups HA5 and HB1 (Graphical abstract) were selected as representative candidates for contact-active and release-active products, respectively, in subsequent evaluations. The systemic toxicity of these materials was assessed following a 30 d subcutaneous implantation in mice. Hematological analysis revealed no significant abnormalities in the counts of white blood cells (WBCs, Figure 4D), lymphocytes (Lyms, Figure 4E) or monocytes (Mons, Figure 4F). Furthermore, biochemical analysis showed that the levels of alanine aminotransferase (ALT, Figure S6A), aspartate aminotransferase (AST, Figure S6B) and creatinine (CREA-S, Figure S6C) remained within normal ranges. These results collectively demonstrate that none of the implanted materials induced systemic inflammation or hematological disorders. Histopathological evaluation of the peri-implant tissues via H&E staining (Figure 4G) showed that all foam variants were encapsulated by a fibrous capsule. Notably, the Mepilex Ag group exhibited a higher degree of inflammatory cell infiltration compared to the HBPL-modified groups, likely triggered by the irritation from dissociated Ag^+^. In contrast, H&E-stained sections of the major organs (heart, liver, spleen, lung and kidney) showed no discernible pathological changes (Figure S6D), suggesting that the toxicity of Ag^+^ remained localized and did not manifest as systemic organ damage within the 30 d period. These findings confirm that the antibacterial foam dressings fabricated via HBPL modification possess excellent biocompatibility, significantly outperforming the conventional silver-containing dressings available on the market.

To further elucidate the disparity in biocompatibility, the extracts were re-prepared using the concentrations in the release antibacterial test to further quantify the cytotoxicity. As shown in Figure 5A, after 24 h of exposure, the Mepilex Ag group remained highly toxic even at a 2-fold dilution, requiring at least a 5-fold dilution to eliminate observable adverse effects. To disclose the underlying mechanism, a series of in-depth cellular assays were conducted. Since a 24 h exposure resulted in complete cell lysis in the Mepilex Ag group, the stimulation time was shortened to 20 min. At this time point, although the cell viability in the Mepilex Ag group had already plummeted to near 0% (Figure 5B), the cellular morphology remained sufficiently intact for fluorescence imaging. Subsequently, a comparative analysis was performed focusing on the intracellular ROS level, mitochondrial membrane potential change, esterase activity and cell membrane permeability, as shown in Figure 5C. The short-term stimulation with the Mepilex Ag extract triggered a profound surge in intracellular ROS, evidenced by intense green fluorescence, a phenomenon absent in all other groups. Furthermore, ΔΨm staining revealed that while other groups maintained normal ΔΨm levels (red fluorescence), the Mepilex Ag group exhibited a shift to green fluorescence, signifying mitochondrial depolarization. The Calcein-AM/PI dual-staining further confirmed these findings. Cells in the Mepilex Ag group were predominantly stained red, indicating compromised membrane integrity and loss of esterase activity, whereas cells in the other groups exhibited robust green fluorescence with negligible red signals. These observations were quantitatively validated by flow cytometry (Figure 5D), where the distinct shift in cell populations for the Mepilex Ag group was readily apparent. Quantitative statistical analysis of the fluorescence intensities (Figure S7A and B) further reinforced these conclusions. Combined with the antibacterial data from the release-killing assays, these results demonstrate that high concentrations of Ag^+^ achieve bactericidal effects at the expense of rapid host cell death. This process is driven by a toxic cascade involving elevated ROS levels, collapsed ΔΨm, diminished esterase activity and increased membrane permeability. In sharp contrast, the HBPL-modified groups (HA5 and HB1) consistently exhibited excellent cytocompatibility, once again highlighting the clinical superiority of HBPL over the conventional silver-based antimicrobial agents.

**Figure 5:**
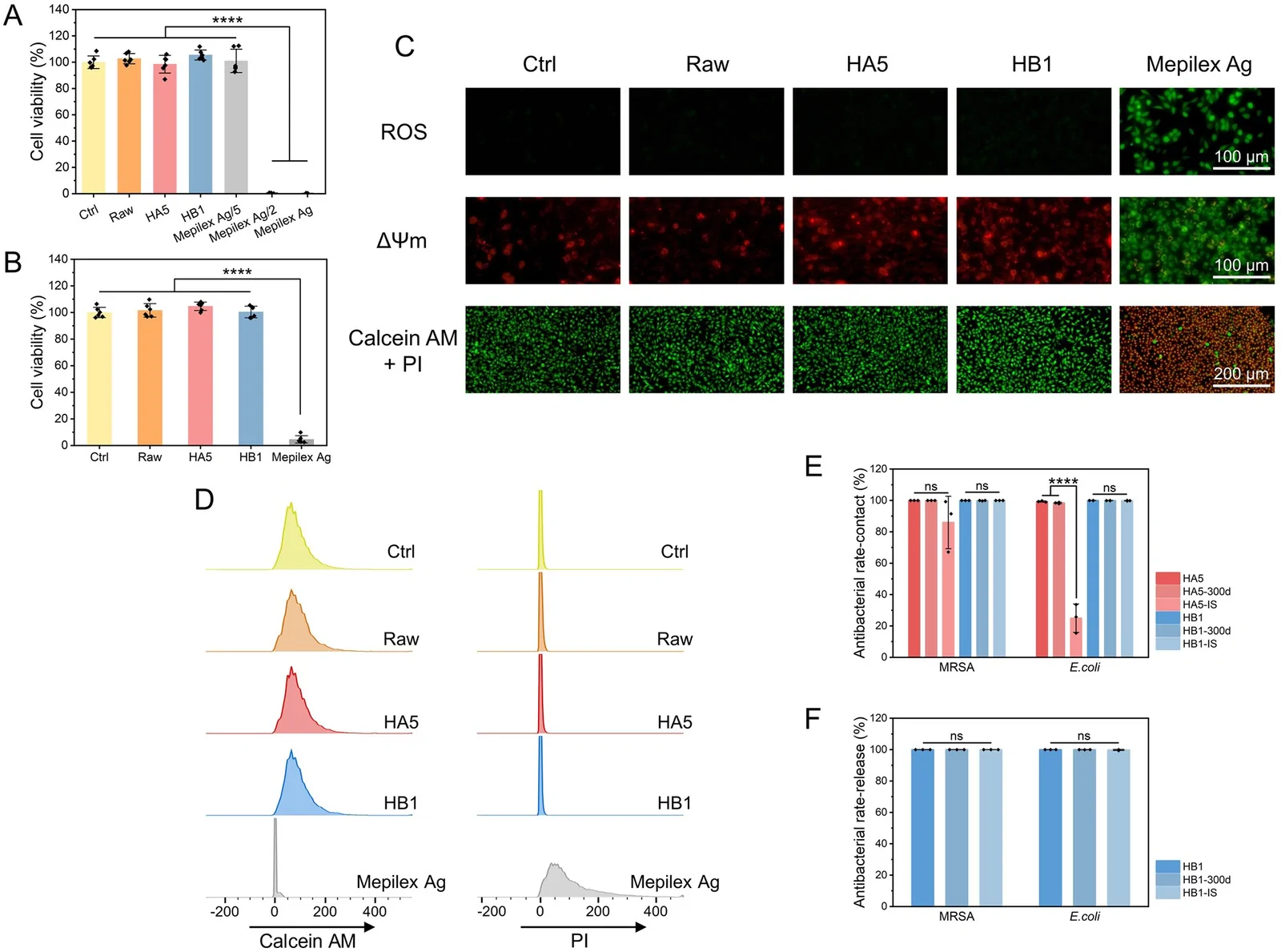
Mechanism exploration of the excellent safety of HBPL-modified foam dressings and their storage and sterilization stability. Viability of L929 cells after being co-incubated with the pure HBPL or Ag of the same concentrations with the foam extracts in the contact antibacterial experiment for (**A**) 1 d and (**B**) 20 min (*n* = 6). (**C**) ROS staining, ΔΨm staining and Calcein AM + PI double-staining photos of each group after stimulation with foam extracts for 20 min. (**D**) Flow cytometry results of Calcein AM + PI double staining. The antibacterial activity of HB1 and HA5 against MRSA and *E. coli*, evaluated using (**E**) contact- and (**F**) release-killing assays after long-term storage (300 d) or irradiation sterilization (*n* = 3). Data are presented as means ± SD. ns represents no significant difference (*P* > 0.05), and **** represents *P* < 0.0001.

### Long-term storage and sterilization verification

To meet the requirements for use as medical devices, the effects of HBPL modification must remain stable during long-term storage and sterilization. To evaluate this stability, packaged HA5 and HB1 samples were either stored at room temperature for 300 days (designated HA5-300d and HB1-300d, respectively) or subjected to irradiation sterilization (designated HA5-IS and HB1-IS, respectively). Changes in their antibacterial activity were then evaluated to determine whether the HBPL modification remained effective.

As shown in Figure S8A and Figure 5E and F, HB1 retained its original antibacterial activity after both 300 days of storage and irradiation sterilization. The antibacterial activity of HA5 changed only slightly after 300 days of storage but declined to some extent following irradiation sterilization. The effects of these two treatments on the total and releasable amino group contents of the two materials are shown in Figure S8B and C. Neither treatment substantially affected their amino group contents. Therefore, the reduced antibacterial activity of HA5 could be attributed to irradiation-induced HBPL chain scission and the resulting decrease in molecular weight. In contrast, the higher HBPL content in HB1 may have rendered this effect negligible.

Overall, HB1 exhibited better compatibility with long-term storage and sterilization processes, making it more advantageous for potential practical applications. For HA5, further optimization of either the sterilization process or the HBPL modification procedure is required to ensure the stability of the modification, or alternatively, the product may be supplied in a non-sterile form.

### Wound healing *in vivo*

A deep burn wound model characterized by excessive exudate was employed to evaluate the pro-healing capacity of the various foam dressings, while MRSA was inoculated to simulate the clinically relevant infection of burn wound *in vivo*. Following the scheme of the animal experimental protocol shown in Figure 6A, the treatment with the foam dressings commenced 1 d post-modeling, with regular dressing changes performed over a total experimental period of 21 d. Representative photographs of the wounds and the corresponding wound closure overlays during the healing process are presented in Figure 6B. By day 6, significant bacterial presence, accompanied by biofilm formation and purulent secretions, was observed in the Ctrl and Raw groups, both of which lacked antibacterial properties. These signs of persistent infection continued until day 15. In contrast, the antibacterial groups (HA5, HB1 and Mepilex Ag) effectively controlled the infection by day 6, characterized by the initiation of scabbing. The quantitative wound healing rates are summarized in Figure 6C. During the early stage of healing (days 0–9), distinct differences were observed among the groups, with the pro-healing efficacy ranking as follows: HB1 ≈ Mepilex Ag > HA5 > Raw > Ctrl. As the infection was gradually brought under control during the intermediate stage, the disparities between groups became narrower. By the study’s endpoint, the wounds in the HA5, HB1 and Mepilex Ag groups were nearly completely closed, whereas the central regions of the wounds in the Ctrl and Raw groups remained incompletely healed.

**Figure 6:**
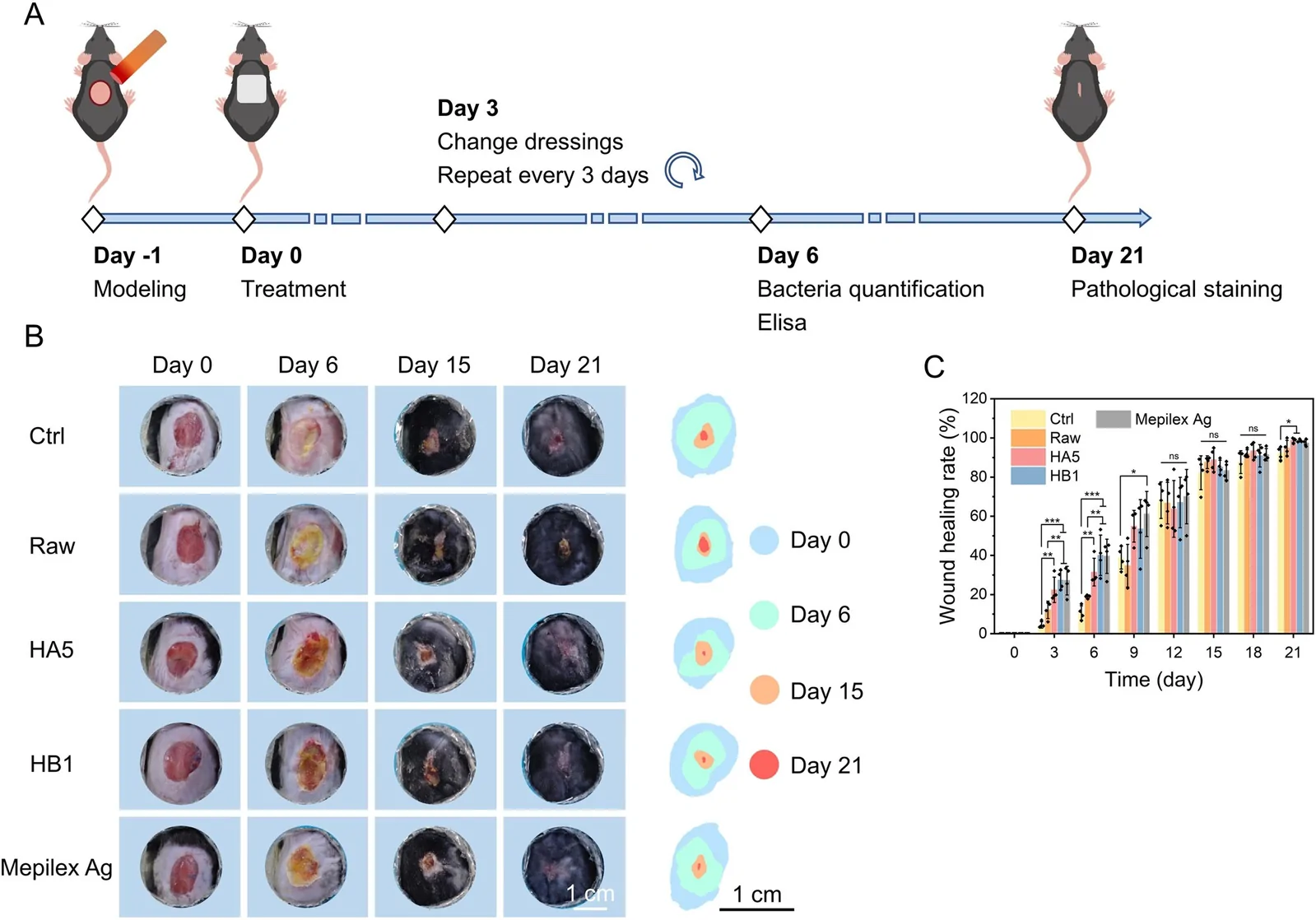
Animal experiment results *in vivo*. (**A**) Flowchart of animal experiments. A 10-mm circular III degree burn wound was created on the back of each C57BL/6 mouse, and infected with 40 μL of MRSA bacterial solution at a concentration of 10^6^ CFU/mL for the modeling at −1 d. (**B**) Representative wound photos and wound overlay images of each group at different time points. (**C**) Wound healing rates at different time points in each group (*n* = 4). Data are presented as means ± SD. *, ** and *** represent *P* < 0.05, 0.01 and 0.001, respectively. *ns* represents no significant difference (*P* > 0.05).

Quantitative analysis of the bacterial burden within the wound tissues on day 6 revealed that the HB1 group possessed the lowest bacterial count, followed by the Mepilex Ag and HA5 groups with marginal increase (Figure 7A and B). In contrast, the Ctrl and Raw groups exhibited high bacterial densities, consistent with the macroscopic observations. Furthermore, the levels of key inflammatory cytokines in the wound tissues were quantified via ELISA. The expression levels of pro-inflammatory cytokines, including tumor necrosis factor-alpha (TNF-α; Figure 7C) and interleukin-1 beta (IL-1β; Figure 7D), were significantly lower in the HA5 and HB1 groups compared to all other groups. The Mepilex Ag group exhibited higher pro-inflammatory cytokine expression than the HBPL-modified groups, despite its potent antibacterial activity. This phenomenon likely stems from the synergistic impact of antimicrobial efficacy and the inherent toxicity of the Ag^+^ components. The irritation caused by released Ag^+^ in the Mepilex Ag group triggered a more pronounced inflammatory response. By contrast, the expression of anti-inflammatory cytokine interleukin-10 (IL-10; Figure 7E) was similar among the HA5, HB1 and Mepilex Ag groups, all of which were significantly higher than those of the Ctrl and Raw groups. These results suggest that while all three types of antibacterial dressings can modulate the immune microenvironment by suppressing infection, the HBPL-modified foams provide a more biocompatible microenvironment with reduced pro-inflammatory stimulation.

**Figure 7:**
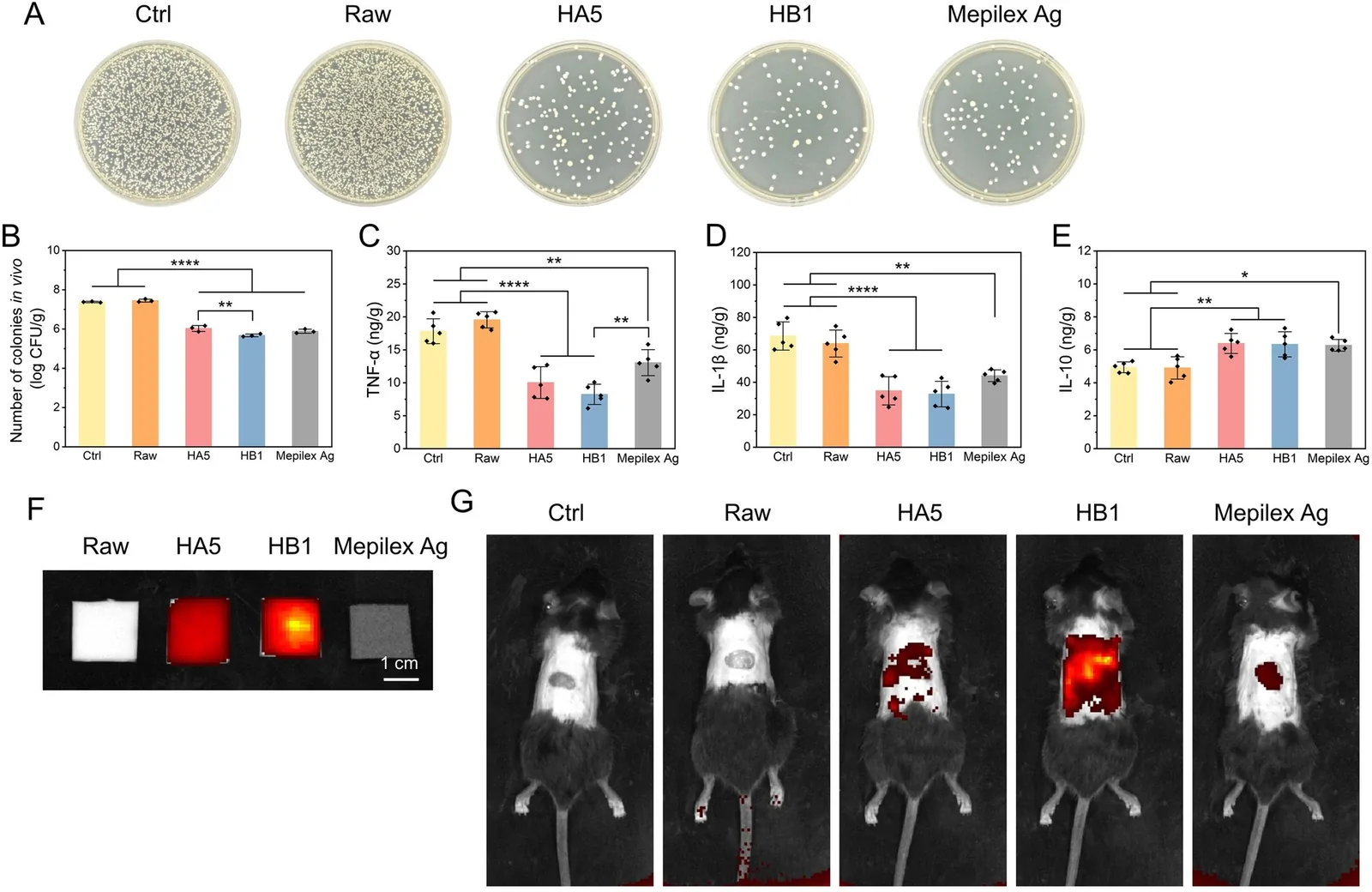
Bacterial remaining and inflammation in animal experiment. (**A**) Colony count photos of bacteria in the wound tissue homogenates of each group at day 6, and (**B**) their quantitative statistics (*n* = 3). the contents of (**C**) TNF-α, (**D**) IL-1β and (**E**) IL-10 tested by ELISA in the supernatant of wound tissue homogenates at day 6 (*n* = 3). (**F**) Fluorescence imaging of foam dressings prepared with fluorescent HBPL, and (**G**) *in vivo* imaging 3 d post their application. Data are presented as means ± SD. *, ** and **** represent *P* < 0.05, 0.01 and 0.0001, respectively.

Furthermore, to investigate the release profile of HBPL *in vivo*, the foam dressings were modified using fluorescently labeled HBPL. As shown in Figure 7F, fluorescence was exclusively observed in groups HA5 and HB1, with group HB1 exhibiting significantly higher intensity due to its higher HBPL loading. Following 3 d treatment, optical imaging *in vivo* was performed (Figure 7G). As expected, no fluorescence was detected in the Ctrl and Raw groups. In group HA5, weak and irregularly distributed fluorescence was observed, which was attributed to the trace amount of residual free HBPL and residual adherence of trace amount of the foam material to the tissue upon dressing removal. For the HB1 group, a distinct and uniform fluorescent signal was observed across the entire contact area, confirming the successful release and diffusion of HBPL into the wound bed. Interestingly, the Mepilex Ag group exhibited faint but detectable fluorescence within the wound region. We hypothesize that this phenomenon arises from the reaction between released Ag^+^ and endogenous reducing agents in the wound environment, such as GSH. This interaction facilitates the reduction and aggregation of silver into specific-sized AgNPs, which possess intrinsic fluorescent properties [42, 43]. This mechanism was successfully replicated *in vitro* by co-incubating Ag^+^ with GSH (Figure S9), providing indirect yet compelling evidence of the high leachability and chemical reactivity of Ag^+^  *in vivo*.

Finally, the microscopic architecture of the wound tissues at the healing endpoint was evaluated via histological sectioning (Figure 8). H&E staining corroborated that the Ctrl and Raw groups still possessed incomplete wound closure, accompanied by extensive inflammatory cell infiltration. The other three groups achieved complete re-epithelialization, but their internal tissue remodeling processes exhibited distinct characteristics. The HB1 group exhibited the minimum inflammatory infiltration and the narrowest unremodeled wound width (Figure S10A). Furthermore, macrophage polarization was analysed via iNOS (M1 marker, green) and CD206 (M2 marker, red) dual-staining. In the HA5 and HB1 groups, the predominant cell population consisted of CD206-labeled M2 macrophages, signifying a transition toward the inflammation resolution phase. Conversely, the Mepilex Ag group, similar to the Ctrl and Raw groups, primarily expressed iNOS-labeled M1 macrophages, indicating persistent chronic inflammation. Masson’s trichrome and Type I (green)/III (red) collagen dual-staining of collagen deposition and maturation revealed the largest area of mature collagen deposition (blue; Figure S10B) and the highest proportion of mature Type I collagen in the HB1 group, whose tissue architecture most closely resembles that of native skin, substantiating the fastest remodeling process. While collagen in the HA5 and Mepilex Ag groups had initiated the transition to Type I, it had not yet organized into robust, thick fibers. In contrast, the Ctrl and Raw groups were still dominated by immature Type III collagen, which serves as a temporary matrix filler. Finally, neovascularization was assessed via CD31 (green) and α-SMA (red) dual-staining. The HB1 group exhibited superior therapeutic outcomes (Figure S10C), characterized by a high density of mature blood vessels co-labeled with CD31 and α-SMA. The remaining four groups primarily contained immature microvessels labeled only with CD31. Collectively, these results demonstrate that the HB1 foam dressing not only accelerates wound closure but also promotes a high-quality tissue regeneration process.

**Figure 8:**
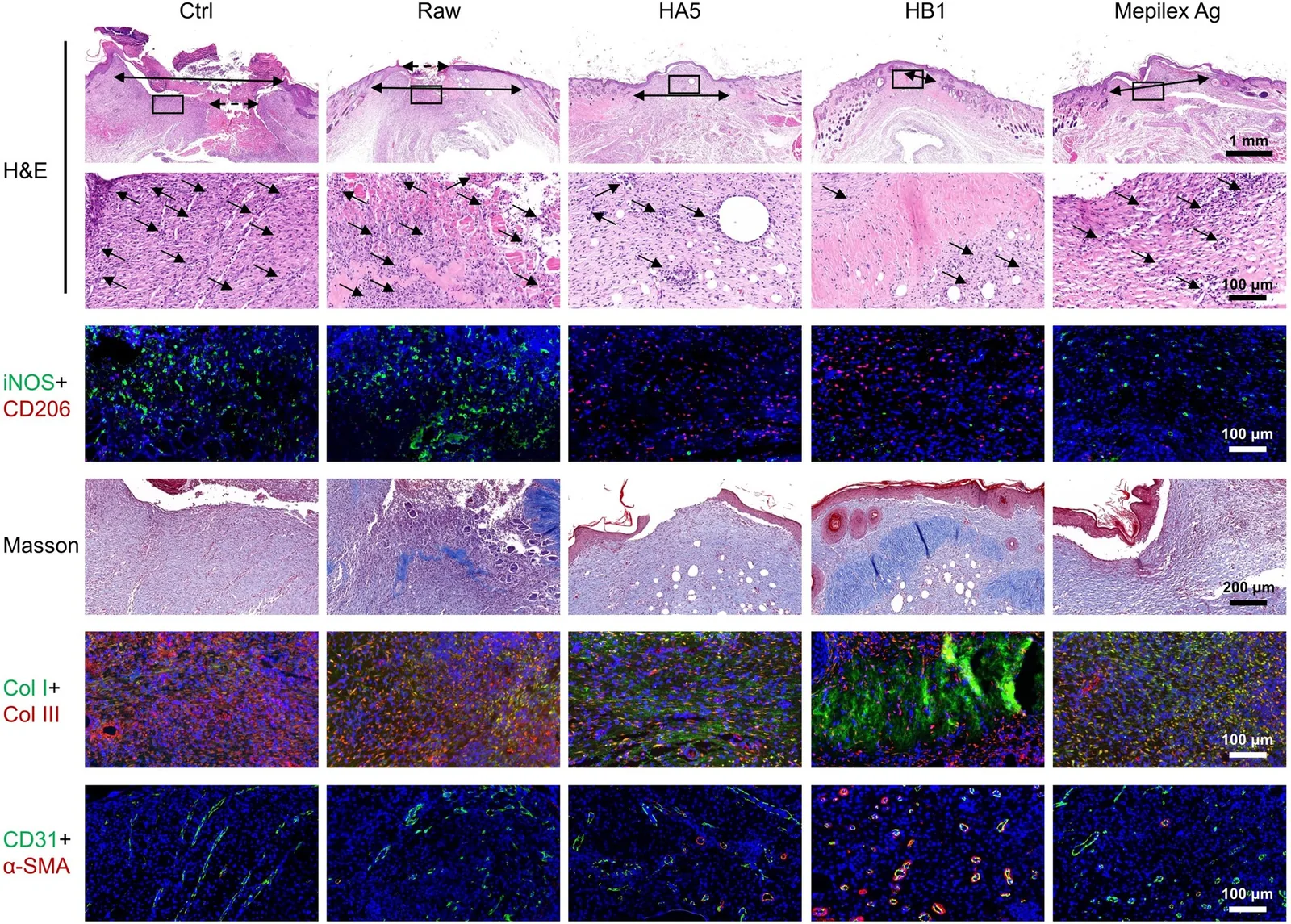
Staining images of tissue sections at the end of healing (day 21). H&E staining, iNOS + CD206 double staining, Masson staining, type I + type III collagen double staining and CD31 + α-SMA double staining images of wound tissues.

In summary, within the context of an MRSA-infected deep burn model, the wound-healing efficacy of the five experimental groups can be distinctly categorized into two tiers: the Ctrl and Raw groups exhibited inferior healing outcomes due to their lack of antibacterial properties, whereas the antibacterial-active groups (HA5, HB1 and Mepilex Ag) significantly accelerated the wound closure rate. Furthermore, owing to the inherent differences in the release kinetics and biocompatibility of the incorporated antimicrobial agents, the HB1 group, utilizing a releasable yet biocompatible HBPL, demonstrated the superior overall healing quality among the active groups.

Rodents such as mice or rats and miniature pigs are common experimental animals in skin injury models. Although miniature pigs are more similar to human skin in terms of skin area and structure, healing mechanism and scar formation [44], herein the widely used mouse model was adopted considering the operational difficulty and experimental cost. It should be acknowledged that due to the inherent constraints of this model (specifically the limited tissue depth), the differences between the HA5 and HB1 groups were not as pronounced as anticipated, despite their vastly different HBPL release profiles. HA5 primarily relies on contact-killing activity, whereas HB1 combines contact-killing with the release of physically adsorbed HBPL. In the present mouse model, the wound was relatively shallow, and the dressing was in close contact with most of the wound bed. Moreover, the highly porous foam could absorb bacteria-containing exudate, bringing bacteria into contact with the HBPL-modified pore surfaces. These characteristics may have reduced the difference between the contact-killing and release-killing mechanisms. In this regard, the release-killing activity of HB1 may be more advantageous in deep, irregular or tunneling wounds, where the dressing cannot directly contact the entire wound bed, whereas the released antimicrobial agents may reach bacteria in non-contact regions. Hence, the advantages of release-active dressings might be displayed in clinical scenarios involving deeper or more complex infected tracks.

## Conclusion

We developed a cost-effective and straightforward modification strategy for PU foam dressings using a novel, high-efficiency and safe antibacterial agent, HBPL. This versatile process ensured stable performance and allowed for a seamless transition between covalently grafted (HyperAnchor series) and free release (HyperBurst series) of HBPL by simply modulating the processing steps. The modification effectively endowed the dressings with potent antibacterial activity without compromising their fundamental physical and mechanical properties. Furthermore, the biocompatibility of the HBPL-modified foams was significantly better than that of the conventional silver-containing dressings (Mepilex Ag). Evaluations *in vivo* further confirmed that these modified foams could coordinate high-quality wound healing in MRSA-infected deep burn models. Given its excellent performance and safety profile, this HBPL-modified foam dressing is poised to be a promising alternative to conventional silver-based dressings for clinical scenarios involving ‘excessive exudate and high infection risk’.

Advanced wound dressings with anti-inflammatory [45, 46], antioxidant [47–49], microenvironment-responsive [50–52], drug-delivery [53, 54], real-time monitoring [55] and/or cell-regulatory [56] capabilities have been widely demonstrated to substantially promote wound healing. In addition to further advancing the industrial scale-up and translation, multifunctional design could be adopted to endow the foam dressing with additional beneficial physiological functions from the perspectives of fundamental studies.

## Supplementary Material

rbag178_Supplementary_Data

## Data Availability

The raw/processed data required to reproduce these findings are available from the corresponding author upon reasonable request.
