# Supplementary material for: Construction of hyperbranched polylysine-modified antibacterial polyurethane foam dressings and their promotion of infected deep burn wound healing
Source: Regen Biomater. 2026 Aug 18;13:rbag178. doi: 10.1093/rb/rbag178 (PMC13637850; doi:10.1093/rb/rbag178)
Supplement: Supplementary file 1 [file rbag178_supplementary_data.docx]

**Supporting Information**

**Construction of hyperbranched polylysine-modified antibacterial polyurethane foam dressings and their promotion of infected deep burn wound healing**

Xiaoqing Liu^#^, Huan Liu^#^, Bingjie Fu, Qiaoju Hu, Min Liang, Xingang Wang^*^, Changyou Gao^*^

**1 Experiments**

**1.1 Materials**

Phosphate-buffered saline (PBS) was purchased from Beyotime Biotechnology (Shanghai, China). AgNO_3_ and glutathione (GSH) were purchased from Shanghai Aladdin Biochemical Technology Co., Ltd. (Shanghai, China). NaOH was purchased from Sinopharm Chemical Reagent Co., Ltd. (Shanghai, China).

**1.2 Cumulative release curves of Ag^+^**

The Mepilex Ag (0.5 g) was immersed in 20 mL of PBS and incubated at 37 °C. Aliquots of the supernatant were collected at predetermined time intervals, and an equal volume of fresh PBS was replenished to maintain a constant total volume. The concentration of Ag^+^ was quantified using an atomic absorption spectrometer (PinAAcle 900T, PerkinElmer, USA). The results were normalized to the dry weight of the foams.

**1.3 Ag^+^ related fluorescence reproduction *in vitro***

To simulate the fluorescence behavior of Ag^+^ in a biological microenvironment, a reaction mixture was prepared by combining 2 mL of GSH (50 mM), 1 mL of Ag^+^ (AgNO_3_, 20 mM), and 17.8 mL of deionized water. The pH of the mixture was adjusted to 7.8 using a NaOH solution. The solution was then incubated in a shaking incubator at 37 °C for 3 d. Following incubation, the fluorescence intensity of the resulting solution was measured using a microplate reader to evaluate the formation of AgNPs.

**2 Supplementary Figures**

**Figure S1.** Total content of HBPL in the foams obtained under three different drying methods, as tested by the ninhydrin method (n=3). Data are presented as means ± standard deviation. *ns* represents no significant difference (p > 0.05).

**Figure S2.** Release curve of Ag^+^ (calculated as Ag_2_SO_4_) in the Mepilex Ag group tested by atomic absorption spectrophotometer (n = 3). Data are presented as means ± standard deviation.

**Figure S3.** Photos of foam dressings before and after modifications at different conditions.

**Figure S4.** Mechanical test data of foam dressings. (A) Tensile strength (n = 6), (B) tensile modulus (n = 6), (C) elongation at break (n = 6), and (D) compressive stress (n = 5). Data are presented as means ± standard deviation. *, ** and *** represent p < 0.05, 0.01 and 0.001, respectively. *ns* represents no significant difference (p > 0.05).

**Figure S5.** Viability of L929 cells after being incubated (8 × 10^3^ per well) with foam extracts (200 μL per well) in a 96-well plate for (A) 1 d, and (B) 3 d (n = 5). (C) Influence of filtration of Mepilex Ag extracts on the viability of L929 fibroblasts (n = 6). Data are presented as means ± standard deviation. *** and **** represent p < 0.001 and 0.0001, respectively.

**Figure S6.** Expression of (A) alanine aminotransferase (ALT), (B) aspartate aminotransferase (AST), and (C) serum creatinine (CREA-S) in the blood 30 d after subcutaneous implantation, where 15 mg foam was implanted for each C57BL/6 mouse, (n = 3). (D) H&E staining images of the main organs after implantation. Data are presented as means ± standard deviation. *ns* represents no significant difference (p > 0.05).

**Figure S7.** Corresponding fluorescence intensity statistics of (A) Calcein AM and (B) PI in the flow cytometry experiment (n = 4). Data are presented as means ± standard deviation. **** represents p < 0.0001.

**Figure S8.** Changes in (A) the antibacterial activity of HB1 and HA5 against MRSA and *E. coli*, evaluated using contact- and release-killing assays, and their (B) relative total amino group content and (C) relative amount of releasable amino groups after long-term storage (300 d) or irradiation sterilization (n = 3). Data are presented as means ± standard deviation. ns represents no significant difference (p > 0.05).

**Figure S9.** Fluorescence generation by Ag^+^ at different conditions *in vitro* (n = 3). Data are presented as means ± standard deviation. **** represents p < 0.0001.

**Figure S10.** Semi-quantitative analysis of (A) the unremodeled wound width in H&E staining, (B) collagen volume fraction in Masson staining, and (C) mature vessel density in CD31/α-SMA immunofluorescence staining (n = 4). Data are presented as means ± standard deviation. *, **, *** and **** represent p < 0.05, 0.01, 0.001 and 0.0001, respectively.
